# Response to Comment on "The role of wildlife (wild birds) in the global transmission of antimicrobial resistance genes"

**DOI:** 10.24272/j.issn.2095-8137.2017.024

**Published:** 2017-07-18

**Authors:** Jing Wang, Zhen-Bao Ma, Zhen-Ling Zeng, Xue-Wen Yang, Huang Ying, Jian-Hua Liu

**Affiliations:** College of Veterinary Medicine, South China Agricultural University, Guangzhou Guangdong 510642, China

## Dear Editor,

Since our first identification of plasmid-mediated colistin resistance gene *mcr-1* in 2015 (Liu et al., 2016), it has been described in human clinics, domestic animals, foods, and the environment worldwide (Schwarz & Johnson, 2016). Although it is still rare, the emergence of *mcr-1* in wild animals is of great concern. We summarized two previous reports on *mcr-1* in wild birds from Lithuania and Argentina to describe its emergence and characteristics in wildlife and highlight the potentially important role of wild animals, particularly birds, in its global transmission (Wang et al., 2017). The first detection of *mcr-1* in wildlife in Asia was identified in an extended-spectrum β-lactamase-producing *Escherichia coli* strain isolated from Eurasian coot (*Fulica atra*), which was located on a ~63 kb IncI2 plasmid, frequently associated with the global transmission of *mcr-1* (Mohsin et al., 2016). The description of *mcr-1* in wild birds in Asia is very important to better understand the role that wild birds may play in the global spread of *mcr-1*, and should have been summarized in our recent review. However, our review only summarized articles published up to December 2016, and as such the then unpublished report on CTX-M-15-producing *Klebsiella pneumoniae* in wild birds in Pakistan (Raza et al., 2017) was not included.

## References

[1] Liu YY, Wang Y, Walsh TR, Yi LX, Zhang R, Spencer J, Doi Y, Tian GB, Dong BL, Huang XH, Yu LF, Gu DX, Ren HW, Chen XJ, Lv LC, He DD, Zhou HW, Liang ZS, Liu JH, Shen JZ. 2016 Emergence of plasmidmediated colistin resistance mechanism MCR-1 in animals and human beings in China: a microbiological and molecular biological study. The Lancet Infectious Diseases, 16 (2): 161- 168. 26603172 10.1016/S1473-3099(15)00424-7

[2] Mohsin M, Raza S, Roschanski N, Schaufler K, Guenther S. 2016 First description of plasmid-mediated colistin-resistant extended-spectrum β-lactamase-producing Escherichia coli in a wild migratory bird from Asia. International Journal of Antimicrobial Agents, 48 (4): 463- 464. 27451084 10.1016/j.ijantimicag.2016.07.001

[3] Raza S, Mohsin M, Madni WA, Sarwar F, Saqib M, Aslam B. 2017 First report of bla_CTX-M-15_-type ESBL producing Klebsiella pneumoniae in wild migratory birds in Pakistan. EcoHealth, 14 (1): 182- 186. 28078492 10.1007/s10393-016-1204-y

[4] Schwarz S, Johnson AP. 2016 Transferable resistance to colistin: a new but old threat. Journal of Antimicrobial Chemotherapy, 71 (8): 2066- 2070. 27342545 10.1093/jac/dkw274

[5] Wang J, Ma ZB, Zeng ZL, Yang XW, Huang Y, Liu JH. 2017 The role of wildlife (wild birds) in the global transmission of antimicrobial resistance genes. Zoological Research, 38 (2): 55- 80. 28409502 10.24272/j.issn.2095-8137.2017.003 PMC5396029

